# Intussusception risk factors and outcomes in adult and elderly patients: analysis from the national inpatient sample

**DOI:** 10.1007/s00423-025-03838-x

**Published:** 2025-10-03

**Authors:** Ben Barris, Guy Elgar, Igor Oliveira, Abbas Smiley, Rifat Latifi

**Affiliations:** 1 Westchester Medical Center, School of Medicine, New York Medical College, Valhalla, NY 10595 USA; 2 University of Rochester Medical Center, Rochester, NY 14642 USA; 3 School of Medicine, University of Arizona, Tucson, AZ 85721 USA

**Keywords:** Intussusception, Elderly patients, Adult patients, Mortality risk, Frailty index, Operative management

## Abstract

**Purpose:**

Intussusception in adult and elderly patients is rare but potentially life-threatening, requiring high clinical suspicion for prompt diagnosis and management. This study aims to identify risk factors in these patients to improve management protocols and enhance clinician awareness.

**Methods:**

Adult and elderly patients with intussusception (2005–2014) from the National Inpatient Sample, which provided data on demographics, comorbidities, outcomes, and operative management were analyzed. Logistic regressions assessed the relationships between mortality and patient characteristics.

**Results:**

Among 4,432 patients, 82.3% were adults (57.1% females), and 17.7% were elderly (64.1% females). The elderly mortality rate was 2.8% versus 0.1% in adults. In non-operatively managed elderly patients, longer hospital length of stays (LOS) and older age increased mortality odds by 5.7% (*P* = 0.120) and 24.8% (*P* = 0.080). Operatively managed elderly patients with higher frailty scores had 34.0% greater mortality odds (*P* = 0.160), while female sex reduced odds by 69.8%. In operatively managed adults, each day of delayed surgery raised mortality odds by 26.7%, and each additional year of age increased odds by 21.0% (*P* = 0.053).

**Conclusion:**

Longer hospital stays, advanced age, and higher frailty increase mortality risk in adult patients and elderly surgical patients. Female sex offers a protective effect. Non-operatively managed elderly patients are at high risk for mortality.

## Introduction

Intussusception is characterized by the telescoping of a proximal segment of the intestine into a distal segment. This process usually occurs due to the entrapment of a lead point by abnormal smooth muscle peristalsis. If not addressed promptly, intussusception can lead to serious consequences, including intestinal obstruction, ischemia, perforation, septic shock, and potentially death [1, 2].

While intussusception is primarily associated with infants and young children, it is a relatively rare entity in adults, accounting for only 5% of all cases of intussusception [1, 2]. The pathogenesis of adult intussusception is multifactorial and complex. Approximately 70–90% of adult cases involve anatomical lesions, including diverticula, polyps, neoplasms, or strictures. These lesions are typically identified during surgery and may act as a lead point, most commonly in the small intestine [1, 2]. Of the anatomical lead points, neoplasms, specifically adenomas, carcinomas, and lymphomas, are most frequently associated with adult intussusception [1, 3]. While intussusception in the large intestine involves a malignant lesion in up to 66% of cases, that of the small intestine has a malignant etiology in up to 30% of cases [2, 4]. Hence, most lead points in the small intestine are benign [4, 5].

The symptoms of adult intussusception are often non-specific, making it difficult to diagnose. Patients may experience abdominal pain, nausea, vomiting, and constipation, all of which are symptoms of many other common gastrointestinal conditions [1, 2]. The similarities in clinical presentations and relative rarity of intussusception in adults, results in reduced clinical suspicion and subsequent delays in clinical intervention [1]. These delays can have severe repercussions, making early diagnosis and prompt treatment critical to reducing adverse patient outcomes [6].

Unlike child intussusception, surgical intervention is most of the time required as the definitive treatment for adult intussusception. Utilization of CT imaging and identification of associated risk factors may help prevent intussusception progression [1, 2].

Previous research has highlighted the morbidity and mortality rates in adults with intussusception. According to previous research, intestinal obstruction is the most common manifestation of adult intussusception and contributes to the increased morbidity that is faced by these patients. In adults diagnosed with intussusception, a mortality rate of 5-7.4% was observed by Traoré et al. [6]. In gathering data from other studies however, mortality rates ranged from 15 to 52.4% [7–10]. Among these investigations, observed mortality rates are highly variable in adult intussusception and indicate the lack of large-scale patient samples. Additionally, mortality rates varied in respect to operational status, malignancy of the lead point, accessibility to screening tools, geographical region, and timeliness of diagnosis.

While etiology and pathophysiology have been well documented in adult intussusception, there has been limited research on the risk factors impacting mortality rates in adults with this condition. Due to the rarity of adult intussusception and the lack of large-scale studies, learning about similar medical conditions can enhance our understanding of this ailment. Drawing from similar medical conditions like bowel obstruction, ventral hernia, and paralytic ileus, mortality risk factors include increased age, increased hospital length of stay (LOS), frailty, delayed time to operation, and male gender [11–14].

This study aims to address the existing knowledge gap by using a large, representative national database to identify patient characteristics predictive of mortality among adults (18–64 years) and elderly (≥ 65 years) patients emergently admitted with intussusception. We hypothesize that advanced age, prolonged hospital length of stay, higher frailty scores, delayed surgical intervention, and male sex will be associated with increased mortality risk.

## Methods

We analyzed data from the National Inpatient Sample (NIS), a comprehensive and nationally representative database sponsored by the Healthcare Cost and Utilization Project (HCUP). We identified all patients aged 18 years or older emergently admitted between 2005 and 2014 with a primary diagnosis of intussusception (ICD-9 code: 560.0). Patients were classified into two groups: adults (18–64 years) and elderly (≥ 65 years). This study included demographic, clinical, procedural, and hospital-related variables available within the NIS.

### Patient characteristics

We have categorized our research data into five separate tables, each stratified according to a different patient characteristic. Both adult and elderly patient groups are represented within the statistics of each table. Tables 1, 2 and 3, which are organized by sex, medical outcomes, and surgical status respectively, contain a plethora of variables from social, economic, and medical domains.

**Table 1:** Characteristics of emergency admitted patients with the primary diagnosis of intussusception (NIS 2005-2014). Data were stratified according to sex categories

		Adult, N (%)			Elderly, N (%)		
		Male	Female	p	Male	Female	p
All Cases		1,563 (42.9%)	2,084 (57.1%)		282 (35.9%)	503 (64.1%)	
Race	White	891 (64.6%)	1,276 (70.0%)	0.008	174 (73.4%)	337 (75.2%)	0.18
	Black	259 (18.8%)	318 (17.5%)		28 (11.8%)	68 (15.2%)	
	Hispanic	159 (11.5%)	149 (8.2%)		19 (8.0%)	20 (4.5%)	
	Asian/Pacific Islander	24 (1.7%)	26 (1.4%)		8 (3.4%)	7 (1.6%)	
	Native American	10 (0.7%)	7 (0.4%)		1 (0.4%)	4 (0.9%)	
	Other	36 (2.6%)	46 (2.5%)		7 (3.0%)	12 (2.7%)	
Income Quartile	Quartile 1	453 (29.5%)	466 (22.8%)	< 0.001	67 (24.3%)	118 (23.8%)	0.98
	Quartile 2	360 (23.5%)	483 (23.7%)		63 (22.8%)	117 (23.6%)	
	Quartile 3	367 (23.9%)	557 (27.3%)		68 (24.6%)	117 (23.6%)	
	Quartile 4	354 (23.1%)	535 (26.2%)		78 (28.3%)	144 (29.0%)	
Insurance	Private Insurance	721 (46.2%)	1,192 (57.3%)	< 0.001	30 (10.6%)	36 (7.2%)	0.28
	Medicare	119 (7.6%)	212 (10.2%)		240 (85.1%)	453 (90.2%)	
	Medicaid	281 (18.0%)	363 (17.4%)		6 (2.1%)	7 (1.4%)	
	Self-Pay	330 (21.2%)	206 (9.9%)		4 (1.4%)	3 (0.6%)	
	No Charge	35 (2.2%)	19 (0.9%)		0 (0%)	0 (0%)	
	Other	73 (4.7%)	89 (4.3%)		2 (0.7%)	3 (0.6%)	
Hospital Location	Rural	142 (9.1%)	190 (9.1%)	0.33	23 (8.2%)	59 (11.7%)	0.16
	Urban: Non-Teaching	582 (37.2%)	825 (39.6%)		121 (42.9%)	189 (37.6%)	
	Urban: Teaching	839 (53.7%)	1,069 (51.3%)		138 (48.9%)	255 (50.7%)	
Invasive Diagnostic Procedure		274 (17.5%)	411 (19.7%)	0.09	52 (18.4%)	61 (12.1%)	0.016
Surgical Procedure		824 (52.7%)	1,164 (55.9%)	0.06	170 (60.3%)	311 (61.8%)	0.67
Invasive or Surgical Procedure		956 (61.2%)	1,359 (65.2%)	0.012	190 (67.4%)	339 (67.4%)	0.995
Deceased		3 (0.2%)	2 (0.1%)	0.66	13 (4.6%)	9 (1.8%)	0.021
		Mean (SD)	Mean (SD)	p	Mean (SD)	Mean (SD)	p
Age, Years		38.41 (13.01)	42.14 (12.06)	< 0.001	75.56 (7.99)	77.79 (8.35)	< 0.001
Modified Frailty Index Score		0.52 (0.79)	0.52 (0.77)	0.99	1.55 (1.10)	1.52 (1.05)	0.72
Time to Invasive Diagnostic Procedure, Days		1.85 (2.33)	1.76 (2.77)	0.67	2.56 (3.97)	2.58 (2.96)	0.97
Time to Surgical Procedure, Days		0.97 (1.96)	0.88 (1.97)	0.32	1.56 (2.83)	1.20 (1.65)	0.1
Length of Stay, Days		4.81 (4.90)	4.87 (5.02)	0.75	7.91 (7.50)	7.36 (5.65)	0.28
Total Charges, Dollars		37,325 (44,066)	37,886 (44,456)	0.71	61,422 (68,368)	57,221 (63,007)	423_2025_3838

**Table 2:** Characteristics of emergency admitted patients with the primary diagnosis of intussusception (NIS 2005-2014). Data were classified according to outcome categories

		Adult, N (%)			Elderly, N (%)		
		Survived	Deceased	p	Survived	Deceased	p
All Cases		3,649 (99.9%)	5 (0.1%)		763 (97.2%)	22 (2.8%)	
Sex, Female		2,802 (57.2%)	2 (40.0%)	0.66	494 (64.8%)	9 (40.9%)	0.021
Comorbidities	AIDS	26 (0.7%)	0 (0%)	0.999	1 (0.1%)	0 (0%)	0.999
	Alcohol Abuse	179 (4.9%)	0 (0%)	0.999	7 (0.9%)	1 (4.5%)	0.2
	Deficiency Anemias	416 (11.4%)	2 (40.0%)	0.1	177 (23.2%)	5 (22.7%)	0.96
	Rheumatoid Arthritis	69 (1.9%)	0 (0%)	0.999	26 (3.4%)	0 (0%)	0.999
	Chronic Blood Loss	41 (1.1%)	0 (0%)	0.999	11 (1.4%)	1 (4.5%)	0.29
	Congestive Heart Failure	46 (1.3%)	0 (0%)	0.999	101 (13.2%)	3 (13.6%)	0.999
	Chronic Pulmonary Disease	412 (11.3%)	2 (40.0%)	0.1	159 (20.8%)	4 (18.2%)	0.999
	Coagulopathy	76 (2.1%)	2 (40.0%)	0.004	23 (3.0%)	3 (13.6%)	0.033
	Depression	383 (10.5%)	0 (0%)	0.999	64 (8.4%)	1 (4.5%)	0.999
	Diabetes, Uncomplicated	215 (5.9%)	1 (20.0%)	0.26	133 (17.4%)	4 (18.2%)	0.999
	Diabetes, Chronic Complications	28 (0.8%)	0 (0%)	0.999	17 (2.2%)	2 (9.1%)	0.1
	Drug Abuse	231 (6.3%)	0 (0%)	0.999	2 (0.3%)	0 (0%)	0.999
	Hypertension	767 (21.0%)	2 (40.0%)	0.28	481 (63.0%)	12 (54.5%)	0.42
	Hypothyroidism	219 (6.0%)	0 (0%)	0.999	132 (17.3%)	3 (13.6%)	0.999
	Liver Disease	101 (2.8%)	2 (40.0%)	0.007	7 (0.9%)	0 (0%)	0.999
	Lymphoma	30 (0.8%)	0 (0%)	0.999	17 (2.2%)	1 (4.5%)	0.4
	Fluid/Electrolyte Disorders	765 (21.0%)	3 (60.0%)	0.07	289 (37.9%)	15 (68.2%)	0.004
	Metastatic Cancer	61 (1.7%)	0 (0%)	0.999	45 (5.9%)	4 (18.2%)	0.043
	Other Neurological Disorders	132 (3.6%)	3 (60.0%)	< 0.001	81 (10.6%)	4 (18.2%)	0.29
	Obesity	201 (5.5%)	1 (20.0%)	0.25	35 (4.6%)	0 (0%)	0.62
	Paralysis	21 (0.6%)	0 (0%)	0.999	9 (1.2%)	2 (9.1%)	0.035
	Peripheral Vascular Disorders	63 (1.7%)	0 (0%)	0.999	71 (9.3%)	3 (13.6%)	0.45
	Psychoses	173 (4.7%)	0 (0%)	0.999	28 (3.7%)	1 (4.5%)	0.57
	Pulmonary Circulation Disorders	22 (0.6%)	0 (0%)	0.999	20 (2.6%)	1 (4.5%)	0.45
	Renal Failure	53 (1.5%)	0 (0%)	0.999	66 (8.7%)	3 (13.6%)	0.43
	Solid Tumor	54 (1.5%)	0 (0%)	0.999	79 (10.4%)	1 (4.5%)	0.72
	Peptic Ulcer	2 (0.1%)	0 (0%)	0.999	2 (0.3%)	0 (0%)	0.999
	Valvular Disease	50 (1.4%)	0 (0%)	0.999	58 (7.6%)	1 (4.5%)	0.999
	Weight Loss	174 (4.8%)	3 (60.0%)	0.001	110 (14.4%)	4 (18.2%)	0.55
Surgical Procedure		1,986 (54.4%)	4 (80.0%)	0.39	461 (60.4%)	19 (86.4%)	0.014
Invasive or Surgical Procedure		2,312 (63.4%)	5 (100%)	0.09	508 (66.6%)	20 (90.9%)	0.019
		Mean (SD)	Mean (SD)	p	Mean (SD)	Mean (SD)	p
Age, Years		40.52 (12.61)	50.80 (13.77)	0.07	76.90 (8.26)	79.77 (8.91)	0.11
Modified Frailty Index Score		0.52 (0.78)	2.00 (1.23)	< 0.001	1.53 (1.07)	1.77 (1.02)	0.29
Time to Invasive Diagnostic Procedure, Days		1.79 (2.60)	8.00 (-)	0.017	2.35 (2.63)	8.00 (11.37)	0.39
Time to Surgical Procedure, Days		0.91 (1.95)	4.67 (3.79)	< 0.001	1.33 (2.15)	1.29 (1.65)	0.95
Length of Stay, Days		4.83 (4.95)	12.80 (8.08)	< 0.001	7.36 (6.09)	14.05 (11.30)	0.012

**Table 3:** Characteristics of emergency admitted patients with the primary diagnosis of intussusception (NIS 2005-2014). Data were stratified according to operation status

		Adult, N (%)			Elderly, N (%)		
		No Operation	Operation	p	No Operation	Operation	p
All Cases		1,664 (45.5%)	1,991 (54.5%)		305 (38.8%)	481 (61.2%)	
Comorbidities	Deficiency Anemias	159 (9.6%)	259 (13.0%)	0.001	73 (23.9%)	110 (22.9%)	0.73
	Chronic Blood Loss	9 (0.5%)	32 (1.6%)	0.002	2 (0.7%)	10 (2.1%)	0.14
	Depression	190 (11.4%)	193 (9.7%)	0.09	37 (12.1%)	28 (5.8%)	0.002
	Diabetes, Uncomplicated	115 (6.9%)	101 (5.1%)	0.019	61 (20.0%)	76 (15.8%)	0.13
	Drug Abuse	121 (7.3%)	110 (5.5%)	0.031	2 (0.7%)	0 (0%)	0.15
	Lymphoma	6 (0.4%)	24 (1.2%)	0.005	8 (2.6%)	10 (2.1%)	0.62
	Other Neurological Disorders	72 (4.3%)	63 (3.2%)	0.06	43 (14.1%)	42 (8.7%)	0.018
	Paralysis	5 (0.3%)	16 (0.8%)	0.045	1 (0.3%)	10 (2.1%)	0.06
	Peripheral Vascular Disorders	16 (1.0%)	47 (2.4%)	0.001	28 (9.2%)	46 (9.6%)	0.86
	Solid Tumor	14 (0.8%)	40 (2.0%)	0.004	18 (5.9%)	62 (12.9%)	0.002
	Weight Loss	54 (3.2%)	123 (6.2%)	< 0.001	37 (12.1%)	77 (16.0%)	0.13
		Mean (SD)	Mean (SD)	p	Mean (SD)	Mean (SD)	p
Age, Years		39.12 (12.63)	41.71 (12.48)	< 0.001	76.74 (8.09)	77.14 (8.41)	0.52
Modified Frailty Index Score		0.50 (0.77)	0.54 (0.78)	0.13	1.52 (1.06)	1.54 (1.08)	0.75
Time to Invasive Diagnostic Procedure, Days		1.88 (1.80)	1.71 (3.20)	0.4	2.07 (1.69)	2.98 (4.33)	0.15
Length of Stay, Days		2.79 (2.37)	6.56 (5.85)	< 0.001	4.50 (5.31)	9.49 (6.24)	< 0.001
Total Charges, Dollars		21,532 (19,852)	51,194 (53,583)	< 0.001	28,198 (33,613)	78,337 (72,291)	< 0.001

Tables 1, 2, 3 and 4, and 5 incorporate the modified frailty index (mFI-5), used to gauge patient’s frailty. This indicator is crucial in clinical practice for the development of targeted interventions and treatment plans, acknowledging the heightened risk of adverse outcomes in frail patients [39]. However, due to the limitations of the NIS database – lack of data on comorbidity duration and functional health status – we estimated mFI-5 scores using available comorbidities.

**Table 4:** Backward logistic regression analysis to evaluate the associations between mortality and different factors in emergency admitted patients with the primary diagnosis of intussusception and undergoing an operation (NIS 2005–2014). Mortality was the dependent variable

	Adult Operation		Elderly Operation	
	N = 1,813	R2 = 0.222	N = 480	R2 = 0.061
	OR (95% CI)	P	OR (95% CI)	P
Time to Operation, Days	1.267 (1.053, 1.525)	0.012	Removed Via Backward Elimination	
Age, Years	1.210 (0.998, 1.466)	0.053		
Sex, Female	Removed Via Backward Elimination		0.302 (0.116, 0.785)	0.014
Modified Frailty Index Score			1.340 (0.894, 2.008)	0.16
Invasive Diagnostic Procedure			Removed Via Backward Elimination	
Race				
Income Quartile				
Insurance				
Hospital Location				

**Table 5:** Backward logistic regression analysis to evaluate the associations between mortality and different factors in emergency admitted patients with the primary diagnosis of intussusception and not undergoing an operation (NIS 2005–2014). Mortality was the dependent variable

	Adult Non-Operation		Elderly Non-Operation	
	N = 1,663	R2 = N/A	N = 305	R2 = 0.219
	OR (95% CI)	P	OR (95% CI)	P
Length of Stay, Days	1 Death		1.057 (0.986, 1.134)	0.12
Age, Years			1.248 (0.977, 1.594)	0.08
Sex, Female			Removed Via Backward Elimination	
Invasive Diagnostic Procedure				
Modified Frailty Index Score				
Race				
Income Quartile				
Insurance				
Hospital Location				

Our estimated mFI-5 score incorporates variables from five categories:

1. Presence of at least one of the following: Solid Tumor, Renal Failure, Metastatic Cancer, Paralysis, Lymphoma, Coagulopathy, or Weight Loss.
2. Diabetes (Uncomplicated) or Diabetes (Chronic Complications).
3. Chronic pulmonary disease.
4. Congestive heart failure.
5. Hypertension (Uncomplicated) or Hypertension (Chronic Complications).

We inferred the presence of diabetes, congestive heart failure, and hypertension if their corresponding comorbidity was indicated. Chronic Obstructive Pulmonary Disease (COPD) was estimated using chronic pulmonary disease comorbidity. As functional health status is absent from the dataset, we had to estimate it based on available variables. If a patient had a comorbidity of a tumor, renal failure, metastatic cancer, paralysis, lymphoma, coagulopathy, or weight loss, we assumed they were functionally dependent.

Both instruments assigned one point to each item if the comorbidity was present. The points were totaled to create an estimated modified frailty score, which ranged from 0 (not frail) to 5 (very frail).

Tables 4 and 5 both utilized a backward multivariate logistic regression with backward elimination for their analysis. Table 4 evaluates the relationships between mortality and various factors in patients with intussusception admitted in an emergency and who underwent surgery. Table 5 explores the associations between mortality and different factors in patients with intussusception admitted as emergencies but who did not undergo surgery.

### Statistical analysis

Numerical data collected was further analyzed utilizing the following statistical methods. Data points pertinent to a specific factor were compounded to calculate the mean, confidence intervals (CI) set at 95%, p-values (0.05 or less considered significant), and standard deviations (SD) throughout the statistical analysis. Categorical and continuous variables were further described through chi-square test and T-test, respectively. Mortality was utilized as a dependent variable. Logistic regression was used to identify predictors of mortality. Initially, univariable logistic regression analyses identified individual associations. Variables demonstrating clinical significance or statistical significance (*p* ≤ 0.05) in univariable analyses were subsequently entered into multivariable logistic regression models. To achieve parsimonious final models, we utilized backward elimination logistic regression, progressively removing non-significant variables (*p* > 0.05) to isolate the strongest mortality predictors. Adjusted odds ratios (OR) and their 95% confidence intervals (CI) were reported.

## Results

### Patient demographics and clinical characteristics

Between 2005 and 2014, a total of 4,432 patients were emergency patients with a primary diagnosis of intussusception, comprising 3,647 adults (82.3%) and 785 elderly patients (17.7%). Females constituted the majority in both adults (57.1%) and elderly (64.1%) groups. The primary insurance type differed by age group: adults primarily had private insurance, whereas elderly patients predominantly had Medicare.

### Comorbidities and mortality

Adults who died exhibited significantly higher prevalence of severe comorbidities, including coagulopathy, liver disease, neurological disorders, and weight loss, compared to adult survivors. In elderly patients, mortality was significantly associated with severe conditions such as coagulopathy, metastatic cancer, fluid/electrolyte disorders, and paralysis, compared to elderly survivors.

### Operative vs. Non-operative Management

Operatively managed adult patients were significantly older and had substantially longer hospital stays compared to non-operative adults (6.6 vs. 2.8 days; *p* < 0.001). Among elderly patients, operative management was associated with significantly longer hospital stays compared to non-operative patients (9.5 vs. 4.5 days; *p* < 0.001). Certain comorbidities, such as neurological disorders, were significantly associated with reduced likelihood of surgical intervention in elderly patients.

Using mortality as the dependent variable, Table 4 demonstrates a backward logistic regression to evaluate the association between mortality and a variety of different factors in patients undergoing an operation. In adult patients, each additional day of delay of our study revealed a remarkable discrepancy in mortality rates among emergency-admitted intussusception patients, with elderly patients exhibiting mortality rates 28 times higher than adults (2.8% vs. 0.1%). operation increased the odds of mortality by 26.7%. While gender was removed as a variable via backward elimination in adult patients, gender played a protective role in elderly patients. Females had a significantly lower decreased mortality, as indicated by the odds ratio of 0.302 (*p* = 0.014).In translating this data point, elderly female patients who underwent an operation were 69.8% less likely to die than males.

In contrast to Tables 4 and 5 illustrates the association between mortality and several different variables in patients not undergoing an operation. Backward logistic regression analysis of conservatively managed adult patients was limited by population size as only 1 death was recorded in the adult non-operative group.

## Discussions

Our study found a significant disparity in mortality rates among emergency-admitted patients with intussusception, with elderly patients experiencing mortality rates 28 times greater than adults (2.8% compared to 0.1%). These findings align with previous reports on gastrointestinal emergencies such as bowel obstruction and colon cancer, where advanced age has consistently been identified as a key mortality predictor [11–13, 15–17].

Longer hospital length of stay (LOS) is widely recognized as a mortality risk factor. Our findings show that deceased adults were hospitalized 7.97 days longer than surviving adults, while deceased elderly patients stayed 6.69 days longer than surviving elderly patients (*p* = 0.012). Table 3 demonstrates that operatively managed elderly patients remained hospitalized 4.99 days longer than their non-operative counterparts (*p* < 0.001). Similarly, operatively managed adults stayed 6.56 days compared to 2.79 days for non-operative adults (*p* < 0.001). Possible contributors include critical illness, post-operative complications, nosocomial infections, and delayed treatment. In accordance with prior research, prolonged LOS is associated with higher mortality [14, 18–20].

Conversely, Lobao et al. reported that extended LOS correlated with lower mortality in elderly patients with ruptured abdominal aortic aneurysms [21]. Additionally, Nath et al. identified a U-shaped pattern in colon cancer, where the highest mortality was seen in both very short (below 7 days) and very long LOS (over 15 days) [2, 22]. In critically ill patients, severe pathology often demands prolonged hospitalization, heightening infection risks and mortality [23]. Adult intussusception commonly originates in the small intestine due to lead points from benign or malignant growths [2, 4, 24–27], with advanced tumors and resultant surgeries contributing to extended LOS. Early detection and intervention are crucial; however, challenges such as obesity, affordability, and limited healthcare accessibility can hinder timely imaging and diagnosis [2, 28–32]. Emphasizing healthy lifestyles, increasing screening opportunities, and addressing socioeconomic barriers through patient/physician education, telemedicine, and healthcare transport services may limit LOS and its associated risks. Future large-scale studies can help clarify additional risk factors for adult intussusception, facilitating earlier recognition, reducing prolonged LOS complications, and ultimately improving outcomes.

Delayed surgery significantly increased mortality in adults with intussusception. Each day of delay raised mortality odds by 26.7% (OR: 1.267, *p* = 0.012). This finding is consistent with previous research on gastrointestinal emergencies like ventral hernia repair and peritonitis management, where timely surgery has been shown to be critical [11, 22, 33]. However, we did not find the same association in elderly patients, likely due to complex factors such as patient candidacy for surgery, reduced physiological reserves, and greater surgical risk overall that comes with age. Nonetheless, we find this to reinforce the need for early diagnosis and management.

Surgical intervention was associated with increased mortality risk in elderly patients (4.0% vs. 1.0% in non-operative management, *p* = 0.014). Elderly patients undergoing intestinal surgery specifically exhibited significantly elevated mortality (3.9% mortality rate), possibly due to the same reasons highlighted above. Our results align with existing literature indicating increased operative mortality among elderly patients undergoing emergent gastrointestinal procedures [11, 15, 17, 22]. Clinicians should carefully weigh surgical risks and benefits in elderly patients.

Our study found nuanced associations between sex and mortality in intussusception. Females made up most of the cases—57.1% in adults and 64.1% in the elderly—and were equally likely to receive surgery. Mortality rates were similar between adult males and females (0.2% vs. 0.1%), but in the elderly, male mortality was more than twice that of females (4.6% vs. 1.8%).

Multivariable analysis showed elderly females had a significantly reduced mortality risk (OR: 0.302, *p* = 0.014), implying they were ~ 70% less likely to die post-operation compared to elderly males. No sex-related mortality differences were found in adults or conservatively managed patients. This difference may relate to comorbidities, as elderly males had higher rates of chronic pulmonary disease, renal failure, and lymphoma. While intussusception prevalence is similar across sexes, slight male predominance has been noted [34]. More research is needed to understand this variability and guide individualized patient care.

## Strengths

Despite the clinical rarity of adult intussusception, our study used a robust dataset of 4,432 patients. The data utilized in our research is highly representative of the adult and elderly population in the United States given its vast data collection across numerous hospitals in the country. The data not only incorporated various medical characteristics but also socioeconomic and demographic factors, leading to conclusions that are generalizable to a wide population. Additionally, the large-scale data outcomes from this study provides us with an unprecedented opportunity to better prognosticate adult and elderly patients with intussusception, potentially improving clinical decision-making. Further research could investigate the risk factors outlined in this study, aiming to reduce mortality, especially among the most vulnerable patient groups.

## Limitations

The dataset that was analyzed did not include any indication of the degree of severity for patient comorbidities. The NIS does not contain all the variables required to calculate the mFI-5, nor does it specify the duration of the comorbidity prior to admission. Due to these limitations, we estimated the mFI-5 with the variables available in the dataset.

Additionally, the NIS does not provide granular definitions or detailed procedural descriptions. This represents an inherent limitation of using administrative databases where clinical detail that would allow for more precise definitions are lacking.

Addressing these limitations could potentially yield more nuanced and accurate data for future studies. Given the retrospective design of this study, there are inherent limitations. Selection bias and unaccounted confounding variables influencing the outcomes cannot be ruled out for certain. Lastly, the backward logistic regression analysis in Table 5 does not display meaningful findings for adults managed without surgery, except for a solitary notation of one death.

## Conclusions

Our study provided more detailed insight into the major risk factors adversely impacting adult and elderly patients emergently admitted with intussusception. In adult patients, hospital length of stay, mFI-5, and time to operation all had a significant association with increased mortality. In elderly patients, hospital length of stay, male sex, and operative management, were among the risk factors that had a connection to increased mortality. These findings reinforce the critical importance of timely diagnosis and management for both adult and elderly patients. However, for the elderly population, detailed frailty and risk assessments should be used to inform careful consideration of surgical risks and benefits. Future studies should investigate and further validate frailty assessments and explore target interventions to improve survival rates in intussusception patients.

## Data Availability

The data supporting the findings of this study are publicly available from the Healthcare Cost and Utilization Project (HCUP) National Inpatient Sample (NIS) database. Access to the data requires registration and approval through the HCUP Central Distributor.
